# Label-Efficient and Lightweight Spectrum Prediction for UAV-Based Spectrum Sensing: A Critical Review

**DOI:** 10.3390/s26175567

**Published:** 2026-09-02

**Authors:** Rong Xu, Changqing Li, Qi Su, Zhangkai Luo, Xianpeng Wang

**Affiliations:** School of Space Information, Space Engineering University, Beijing 101416, China; 17813205220@163.com (R.X.); suqi@hgd.edu.cn (Q.S.); luo_zhangkai@126.com (Z.L.); 17889944976@163.com (X.W.)

**Keywords:** UAV, spectrum prediction, label-efficient learning, few-shot learning, lightweight deployment

## Abstract

Radio-spectrum prediction can support proactive channel verification, sensing scheduling, and access decisions in unmanned aerial vehicle (UAV) systems. However, existing evidence remains fragmented across UAV-oriented prediction, label-efficient learning, and deployment-oriented efficiency. This article presents a structured critical review of studies identified in IEEE Xplore, Scopus, and the Web of Science Core Collection from database inception to 24 August 2026. Studies were included when they evaluated the prediction of a future spectrum-related condition and were excluded when they addressed only current-state sensing, static spectrum mapping, UAV detection or classification, localization, or superseded study versions. The final corpus comprised 76 retained publications, including 63 original studies and 13 background references. The original studies included 14 direct UAV-related prediction studies, 38 transferable radio-spectrum studies, and 11 UAV-scenario studies. The review shows that transfer learning currently provides the most consistent support for reducing target-domain data requirements when related source bands, sensing stations, or radio environments are available. Self-supervised and other unlabeled-data methods are particularly relevant to UAV missions that can continuously collect spectrum traces but cannot obtain extensive labels, whereas meta-learning remains promising but lacks a standardized support–query evaluation protocol for UAV spectrum prediction. Generative augmentation can expand limited training data, but its effectiveness depends on whether the generated samples preserve the temporal, spectral, spatial, and propagation characteristics of the target environment. Lightweight architectures, online learning, model compression, knowledge distillation, FPGA implementation, and embedded execution provide complementary efficiency mechanisms, but their benefits should be distinguished from one another. Embedded prediction-related processing has been demonstrated on Raspberry Pi and software-defined-radio platforms; however, no end-to-end validation of a UAV-mounted predictor during flight was identified. Overall, the evidence suggests a conditional trade-off among target-domain data requirements, prediction generalization, adaptation cost, and deployment efficiency rather than a universal conflict between few-shot learning and lightweight models. The principal research gap is the limited joint validation of UAV-acquired data, target-domain adaptation, efficient inference, uncertainty-aware decision making, and onboard hardware under representative flight conditions.

## 1. Introduction

Radio spectrum prediction estimates future radio-spectrum conditions from historical measurements and contextual information. Unlike spectrum sensing, which estimates the current state of a frequency band, spectrum prediction provides advance information about spectrum occupancy, availability, received signal strength (RSS), power spectral density (PSD), state duration, or time–frequency activity. This information can support proactive channel selection, adaptive sensing scheduling, handover preparation, and dynamic spectrum access. Early studies and reviews classified spectrum prediction according to statistical models, machine-learning methods, and deep-learning architectures, while subsequent work emphasized temporal, spectral, spatial, and joint multidimensional dependencies in spectrum observations [1,2,3,4].

The highly dynamic operating environments of unmanned aerial vehicles (UAVs) and low-altitude communication systems pose forecasting challenges that are not adequately captured by conventional fixed-location approaches. During the interval between sensing, prediction, and decision execution, a UAV may move to a new position where historical spectrum observations are sparse or unavailable. Variations in altitude, trajectory, line-of-sight conditions, antenna orientation, traffic activity, propagation environment, and interference can also change the distribution of received spectrum measurements. Consequently, a model trained at one position or during one observation period may not remain reliable at another position or at a later stage of a mission. UAV-oriented spectrum prediction therefore involves not only temporal forecasting but also next-location inference, missing spatial history, target-domain variation, and mobility-induced distribution shift [5,6,7,8,9,10].

Research on UAV-related spectrum prediction has progressively expanded beyond fixed-location temporal forecasting. Homotopy- and hidden-Markov-model-based approaches addressed the prediction of spectrum states at future positions when direct historical observations at those positions were unavailable [6,7,8,9,10,11]. Other studies investigated UAV-related spectrum-state prediction, three-dimensional spatial prediction, low-altitude spectrum prediction, and future frequency-hopping patterns [11,12,13,14,15,16,17,18]. A separate line of research predicted time–frequency activity associated with UAV radio-frequency (RF) signals [19].

These developments expanded both the prediction target and the role assigned to the UAV. Depending on the study, the UAV may act as a communication node, sensing platform, computing node, RF emitter, or prediction target. These roles imply different data sources, validation conditions, and deployment assumptions and should therefore be distinguished when interpreting the evidence. In particular, prediction of a UAV-emitted RF signal is different from prediction of the spectrum available to a moving UAV. Neither setting alone establishes that the prediction model was executed on UAV-mounted hardware during flight.

UAV spectrum sensing, three-dimensional spectrum measurement, radio-environment mapping, and aerial channel-map construction offer complementary insights into altitude, spatial coverage, sampling density, trajectory, and data-acquisition constraints [20,21,22,23,24,25,26,27,28,29,30]. These studies are important for understanding the data required by a mobile spectrum-prediction system. However, current-state sensing, spatial reconstruction, and spectrum mapping should not be taken as direct evidence of future predictive capability unless the study explicitly evaluates a future spectrum condition.

A related body of radio-spectrum research has investigated how prediction models can operate when labeled target-domain data are limited. Cross-band transfer learning, GAN-based augmentation, meta-learning, self-supervised representation learning, cross-domain knowledge distillation, and incomplete-observation learning address this problem through different mechanisms [31,32,33,34,35,36,37,38,39,40,41,42,43]. These approaches are often discussed together, but they should not all be classified as few-shot learning. In its strict formulation, few-shot learning uses a small labeled support set for rapid adaptation and evaluates generalization on a separate query or test set. Transfer learning reuses knowledge from a source domain, domain adaptation addresses source–target distribution differences, and self-supervised learning derives supervisory information from unlabeled observations. Accordingly, this review treats few-shot learning as a subset of the broader category of label-efficient learning.

This distinction is particularly important in UAV applications. A small dataset may reflect incomplete spatial coverage, a short observation period, or a limited number of labeled samples, but these conditions are not interchangeable. Cross-band transfer learning has been used to address limited target-domain history [31]. GAN-based methods have generated additional target-like spectrum observations [32,35]. Transfer learning has also been combined with meta-learning for small-data adaptation [33], while deep three-dimensional prediction and temporal–frequency fusion have been investigated using broader radio-spectrum datasets [34,36,38]. Cross-domain knowledge distillation has transferred information from a data-rich station to a data-limited station [39]. Self-supervised graph learning, tensor-based reconstruction, untrained deep priors, and federated radio-map estimation have further explored the use of unlabeled or incompletely observed spectrum data [37,40,41,42,43].

Onboard deployment introduces a second dimension of efficiency. Lightweight architectures, online learning, model compression, quantization, knowledge distillation, and cloud–edge collaboration can reduce different components of deployment cost [44,45,46,47,48,49,50,51,52,53,54,55,56,57,58,59,60,61,62,63,64,65,66,67]. Nevertheless, reduced training time, reduced sensing frequency, or offloaded computation does not necessarily mean that the prediction model itself is lightweight. Model efficiency concerns parameter count, storage size, computational operations, memory footprint, inference latency, and energy per inference. System efficiency additionally includes sensing overhead, communication cost, model-update cost, power consumption, energy use, and mission-level effects.

The existing evidence illustrates this distinction. Dictionary learning, recurrent-model simplification, fast LSTM initialization, broad learning, graph learning, online sequential learning, and knowledge distillation have been used to reduce computation or adaptation cost [51,52,53,54,55,56,57,60,61,62,63,64,65,66,67]. Embedded prediction-assisted spectrum sensing has been implemented on Raspberry Pi 5 and software-defined radios [49]. FPGA implementation has provided evidence for real-time RF spectral prediction [50]. Quantization-aware spectrum sensing on a resource-constrained processor has demonstrated hardware-oriented model optimization, but its task was current-state sensing rather than future spectrum prediction [56].

Previous surveys have provided broad classifications of spectrum-prediction algorithms, data sources, evaluation metrics, spectrum mapping, and spectrum inference [1,2,3,4,68,69,70,71,72,73,74,75]. UAV-related studies have separately examined the integration of cognitive radio with UAVs, three-dimensional spectrum sensing, radio-environment mapping, volumetric measurements, and aerial channel knowledge maps [5,20,21,22,23,24,25,26,27,28,29,30]. However, these bodies of literature have not always been synthesized under a common evidence framework. Existing reviews provide limited comparative analysis of whether a method was evaluated in a direct UAV-related prediction task, demonstrated only on terrestrial spectrum data, or inferred from adjacent sensing and deployment studies. Information on whether reductions in target-domain data requirements were accompanied by reductions in model size, computation, latency, memory, energy consumption, or hardware burden has also been reported inconsistently.

This review addresses this gap by examining UAV-oriented spectrum prediction from three related perspectives: the evolution and operating constraints of UAV-based prediction tasks, label-efficient methods that reduce target-domain data requirements, and lightweight or deployment-oriented methods that reduce model and system cost. Generalization and compactness are treated here as a trade-off that depends on the task and deployment context, rather than as a fixed opposition. A larger model is not necessarily more transferable, and a compact model does not necessarily generalize poorly. Their relationship depends on the prediction target, source–target similarity, adaptation strategy, environmental variation, update protocol, and target hardware.

The main contributions of this review are as follows:

1. We clarify the task and terminology boundaries of UAV-oriented spectrum prediction by adopting explicit working distinctions among current-state spectrum sensing, future spectrum prediction, static spectrum mapping, next-location prediction, and UAV-associated frequency-hopping prediction. Strict few-shot learning is also distinguished from the broader category of label-efficient learning.
2. We provide a structured reconstruction of the field’s development by tracing changes in prediction targets, spatial and temporal scope, operating conditions, and data-efficiency requirements. The review covers the progression from next-location and time–frequency prediction to spectrum-state duration estimation, arbitrary-flight-path prediction, three-dimensional and low-altitude prediction, sparse-data adaptation, and frequency-hopping prediction.
3. We develop a common framework for assessing evidence relevance and deployment realism. Direct UAV-specific prediction evidence is distinguished from transferable evidence from general radio-spectrum studies and from evidence concerning UAV operating contexts. The framework separately records the UAV’s role, data provenance, validation setting, hardware platform, and degree of deployment realism.
4. We identify a central gap in the existing evidence base. Although prior studies have addressed UAV-related prediction, target-domain data scarcity, computational efficiency, embedded execution, and spectrum-drift adaptation, the existing literature provides limited integrated evidence on their joint satisfaction using UAV-acquired data, UAV-mounted hardware, and representative flight conditions.

The remainder of this article is organized as follows. Section 2 describes the review scope, search process, screening procedure, and evidence organization. Section 3 defines the prediction tasks and summarizes the development and constraints of UAV-oriented spectrum prediction. Section 4 examines label-efficient learning methods. Section 5 reviews lightweight and deployment-oriented approaches. Section 6 provides a comparative synthesis and outlines future research priorities. Section 7 concludes the review.

## 2. Review Scope and Methodology

### 2.1. Review Scope

This review considered methods for predicting a future radio-spectrum condition, signal-related quantity, spectrum-state duration, time–frequency pattern, or spatial spectrum condition in UAV-related, low-altitude, or transferable radio-spectrum settings.

The literature search covered IEEE Xplore, Scopus, and the Web of Science Core Collection. Three related research directions were considered: UAV- and low-altitude-oriented spectrum prediction, label-efficient radio-spectrum prediction, and lightweight or deployment-oriented spectrum prediction. This scope reflects the combined requirements of UAV spectrum prediction, including the need to model changing radio environments while operating with limited data and onboard computational resources.

**Figure 1:**
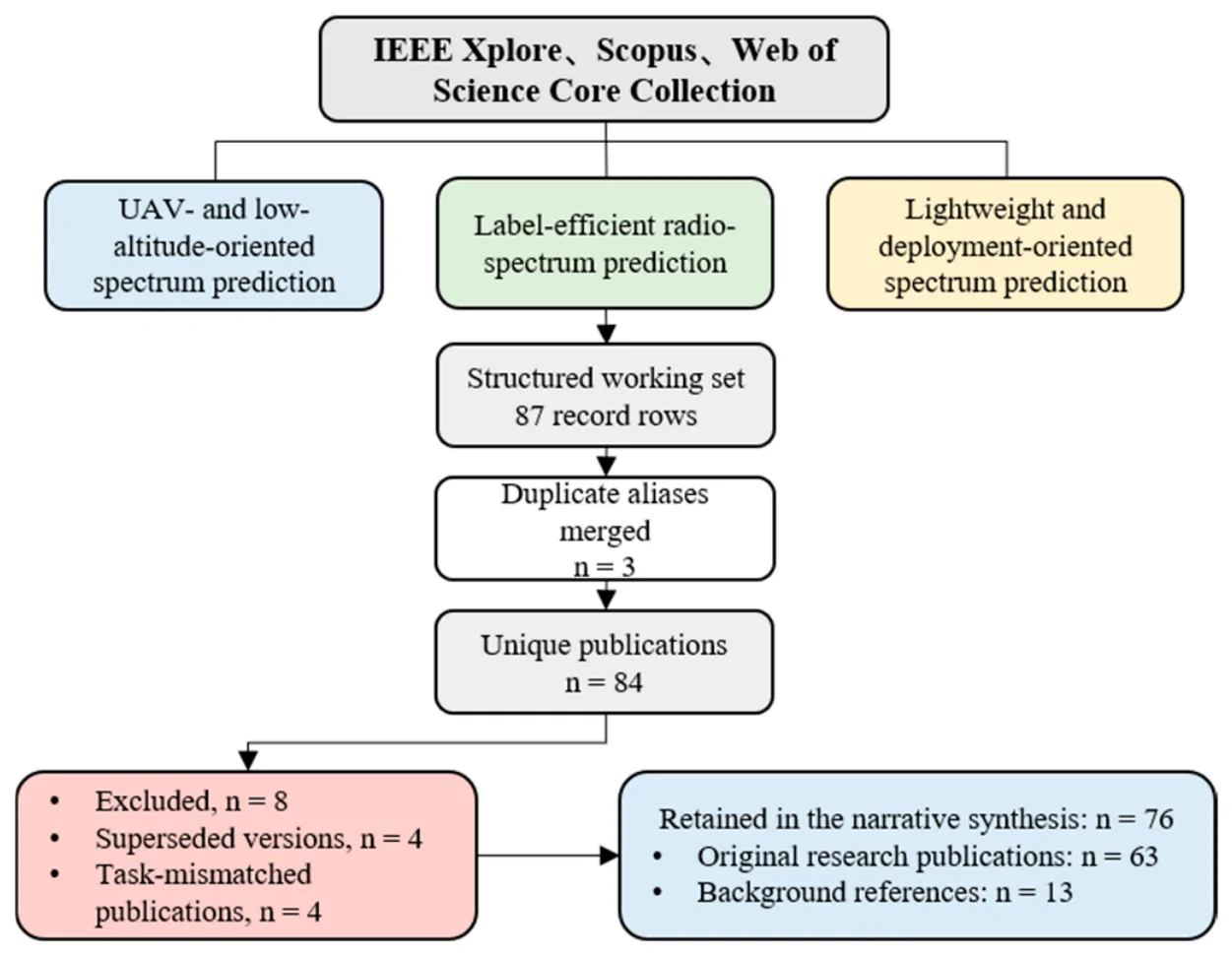
Literature-search and screening workflow.

Figure 1 presents the literature-search and screening workflow. The structured working set contained 87 records. After removing three duplicate records identified across databases, 84 unique records remained. Eight records were excluded from the final synthesis, including four earlier versions superseded by later publications and four publications addressing UAV detection, classification, tracking, or localization rather than future spectrum prediction. Accordingly, 76 publications were retained for the narrative synthesis, comprising 63 original research publications and 13 background references.

### 2.2. Evidence Classification and Extraction

The retained publications were classified into four evidence categories according to their relationship to the review topic:

- Core/E1: direct UAV- or low-altitude-oriented spectrum-prediction studies, including UAV-associated RF prediction;
- Adjacent-A/E2: transferable radio-spectrum prediction, adaptation, efficiency, or deployment methods evaluated without direct UAV onboard validation;
- Adjacent-B/E3: UAV spectrum sensing, measurement, mapping, sampling, channel knowledge, or deployment-constraint studies relevant to spectrum prediction;
- Background/E4: reviews, tutorials, and methodological studies used for terminology and conceptual classification.

This classification distinguishes direct UAV evidence from technically relevant evidence obtained in general radio-spectrum settings. The presence of a UAV in a study was therefore not, by itself, sufficient for classification as Core/E1. The final evidence classification is summarized in Table 1.

## 3. UAV Spectrum Prediction: Tasks, Development, and Constraints

### 3.1. Prediction Tasks in UAV-Based Spectrum Sensing

**Figure 2:**
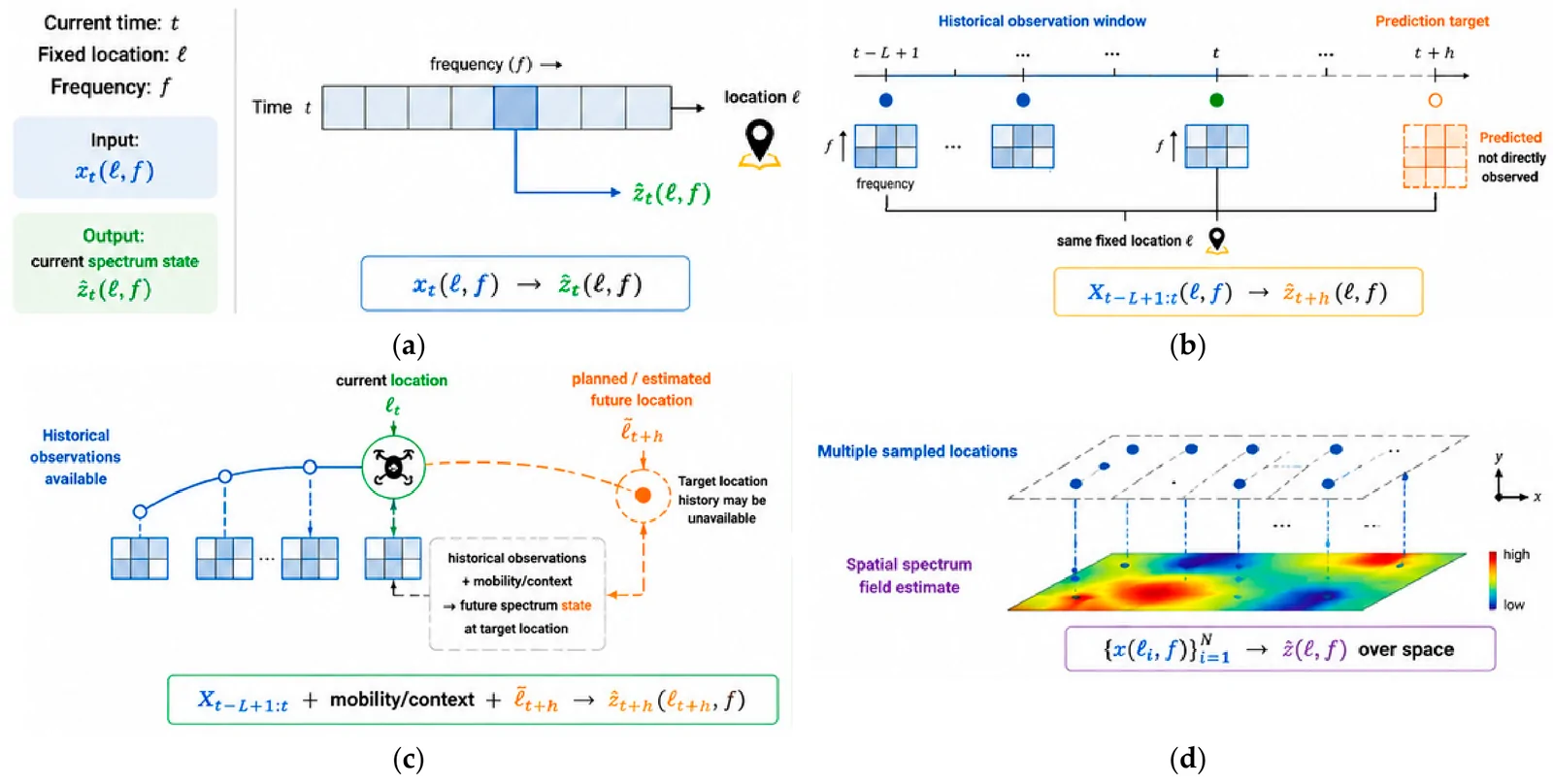
Conceptual boundaries among spectrum sensing, spectrum prediction, and spectrum mapping: (**a**) Estimation of the current spectrum condition at a fixed location; (**b**) Prediction of a future spectrum condition at the same location, with green dots indicating data points that are not directly observed but inferred or predicted; (**c**) Prediction at the planned or estimated future location of a UAV; and (**d**) Reconstruction of a spatial spectrum field from measurements collected at multiple locations.

Spectrum sensing, spectrum prediction, and spectrum mapping are closely related but distinct tasks. Spectrum sensing estimates the current radio state from observations collected at time t, whereas spectrum prediction estimates a future spectrum state, signal value, state duration, or frequency pattern [1,2]. Spectrum mapping reconstructs the spatial distribution of a spectrum-related quantity from measurements collected at multiple locations [20,23,24,25,26,27]. Figure 2 illustrates these task boundaries and highlights the transition from current-state sensing to fixed-location temporal prediction and mobility-aware UAV prediction.

As shown in Figure 2a, current-state spectrum sensing estimates the spectrum condition at the observation time and location, without introducing a future prediction horizon. To establish a common notation for the reviewed studies, current-state spectrum sensing can be represented in an abstract form as [1,2]:

(1) $${\hat{z}}_{t}\left(l,f\right)=g_{\phi }\left(x_{t}\left(l,f\right)\right),$$

where $x_{t}\left(l,f\right)$ denotes an observation collected at time t, location l, and frequency f, and ${\hat{z}}_{t}\left(l,f\right)$ is the estimated current spectrum state or signal quantity.

In fixed-location prediction, illustrated in Figure 2b, historical observations are used to estimate the spectrum condition at a future time while the target location remains fixed. This task can be represented as

(2) $${\hat{z}}_{t+h}\left(l,f\right)=f_{\theta }\left(X_{t-L+1:t},\left(l,f\right)c_{t-L+1:t}\right),$$

where L  denotes the observation-window length, h denotes the prediction horizon, X  contains historical spectrum observations, and c  denotes the available contextual variables.

UAV next-location prediction extends fixed-location prediction by allowing the target location to change together with the prediction time, as illustrated in Figure 2c. Based on the settings considered in [6,7,8,9,10,11], UAV next-location prediction can be represented as

(3) $${\hat{z}}_{t+h}\left(l_{t+h},f\right)=f_{\theta }\left(X_{t-L+1:t},l_{t},{\tilde{l}}_{t+h},m_{t:t+h},c_{t-L+1:t+h}\right),$$

where ${\tilde{l}}_{t+h}\;$ denotes the planned or estimated future location and $m_{t:t+h}$ denotes available mobility information, such as altitude, velocity, heading, waypoints, or relative displacement. In this setting, historical measurements may be unavailable at the target location, making spatial transfer and mobility information relevant to the prediction process.

Equations (1)–(3) are review-level abstractions introduced to provide a common description of the three task settings. They are not intended to reproduce the exact model structures or input variables of any single study.

Spectrum mapping, shown in Figure 2d, differs from these temporal tasks because it reconstructs a spatial field from observations collected at multiple locations and does not necessarily involve a future horizon. Accordingly, spatial interpolation or reconstruction is treated as spectrum prediction only when the target explicitly concerns a future spectrum condition.

Based on these distinctions, the prediction targets were grouped into five categories:

- Spectrum-state or occupancy prediction: prediction of whether a channel will be occupied or available at a future time or position [6,7,8,9,10,11,12,14];
- Spectrum-state duration prediction: prediction of how long an idle or occupied state will persist [10];
- RSS or PSD prediction: regression of future signal strength or power spectral density [36,38,54,76];
- Future spatial spectrum prediction: estimation of future or unobserved spectrum values over two- or three-dimensional regions [13,20,34,36,38]; and
- Frequency-hopping or UAV-associated RF prediction: prediction of future hopping frequencies, time–frequency points, or hopping sequences [15,16,17,18,19].

These tasks should not be combined in a single numerical ranking because they differ in target variable, spatial scope, prediction horizon, class distribution, and evaluation protocol. Prediction probability, classification accuracy, prediction-feasibility ratio, MAE, RMSE, MAPE, and occupancy-rate error quantify different aspects of performance.

In particular, a result obtained from fixed-location temporal prediction should not be treated as direct evidence of next-location prediction. Similarly, prediction of an RF signal associated with a UAV does not by itself demonstrate prediction of the spectrum available to a moving UAV or execution of the predictor onboard.

### 3.2. Prediction-Assisted Sensing Workflow

Spectrum prediction supports proactive sensing and access decisions, but it does not eliminate the need for physical sensing. Historical inputs may include channel-occupancy sequences, RSS or PSD measurements, sensing locations, UAV trajectories, and other contextual variables. The predicted future spectrum condition can be used to prioritize channels for verification, adjust sensing intervals, or reduce the sensing search space [1,2,35,36,57].

**Figure 3:**
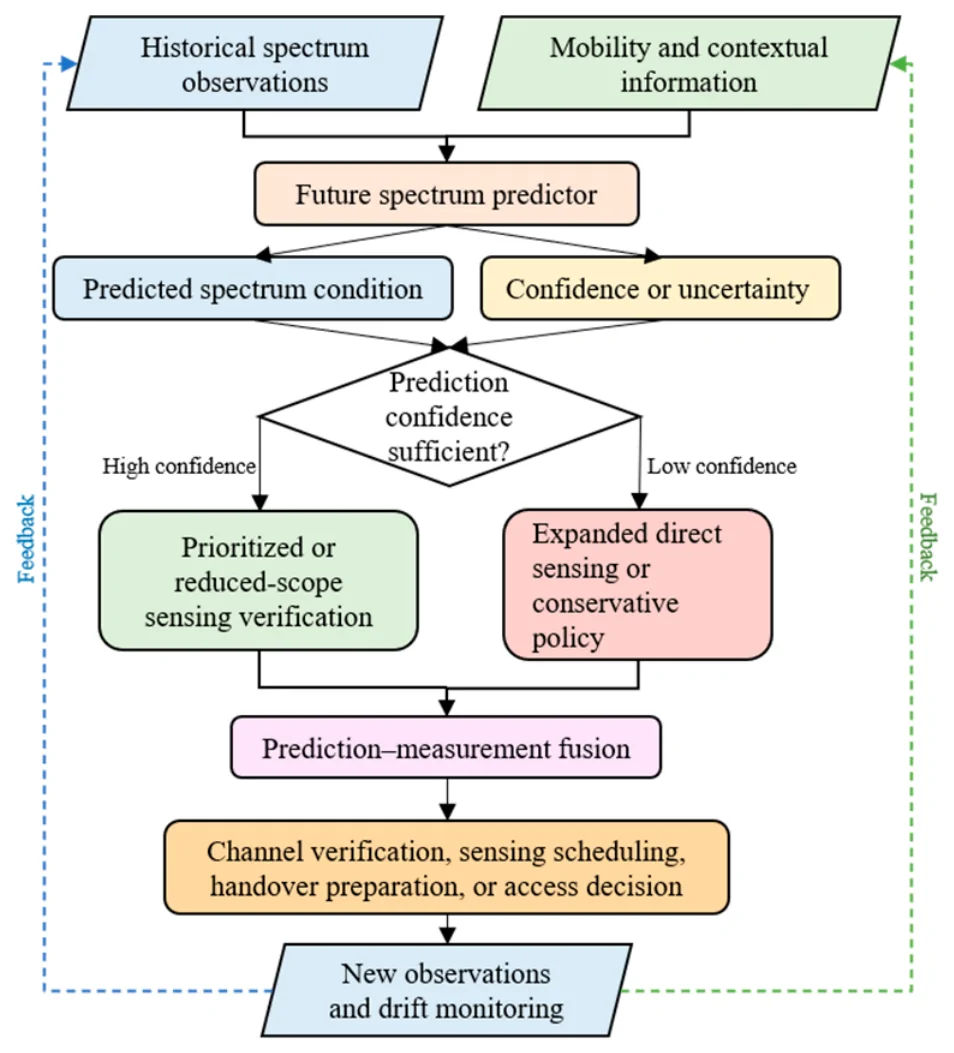
Confidence-aware prediction-assisted UAV spectrum-sensing workflow.

The general role of prediction within a confidence-aware sensing and decision loop is illustrated in Figure 3.

As shown in Figure 3, historical spectrum observations are processed together with mobility and contextual information by a future spectrum predictor. The predictor produces an estimated spectrum condition at a future time, frequency, or UAV position, together with a confidence or uncertainty measure. The confidence estimate determines how the prediction is used in the subsequent sensing process.

When the prediction confidence is sufficiently high, the system may prioritize a subset of channels or reduce the sensing scope before direct verification. When the confidence is low, the system should expand direct sensing or adopt a conservative access policy. Thus, prediction functions as a prior for sensing and decision making rather than as a replacement for measurement. New observations are subsequently incorporated to monitor prediction drift and update the sensing strategy.

Incorrect predictions may lead to missed access opportunities, unnecessary channel switching, repeated sensing, retransmissions, or interference with incumbent users. These risks motivate the use of confidence-aware policies in which the degree of sensing reduction depends on prediction reliability.

Single-step prediction is mainly relevant to immediate channel preselection and short-term sensing adjustment. Multi-step prediction may additionally support handover preparation, sensing-resource allocation [13,34,38], and trajectory planning. However, prediction reliability should be reassessed as new observations become available because uncertainty generally tends to increase with the prediction horizon.

For next-location prediction, errors in the planned or estimated UAV position may alter the expected spectrum condition at the target location. Performance should therefore be evaluated under realistic mobility-information errors rather than only under perfectly known trajectories.

A reduction in sensing frequency should be interpreted as a system-level benefit, rather than as direct evidence that the prediction model is lightweight. Practical evaluation should jointly consider prediction accuracy, selective-sensing effectiveness, access reliability, computational cost, and energy consumption.

### 3.3. Constraints in Low-Altitude UAV Scenarios

UAV spectrum prediction is affected by target-domain data scarcity, spatiotemporal variation, sensing uncertainty, and limited onboard resources.

A moving UAV may reach a location for which no historical spectrum sequence is available. Measurements obtained at nearby positions may not be directly transferable because altitude, propagation loss, line-of-sight probability, antenna orientation, interference, and radio traffic may differ [7,11,22,23,24,25,26,27,28,29,30]. Data scarcity may therefore involve incomplete spatial coverage, missing altitude levels, absent frequency bands, irregular observations, corrupted measurements, and imbalance between occupied and idle states.

Representative flight data require synchronized spectrum, position, altitude, velocity, heading, and trajectory information. UAV-based measurement and mapping studies have demonstrated the feasibility of three-dimensional or volumetric acquisition [23,24,25,26,27]. Nevertheless, map reconstruction and measurement do not independently establish future spectrum prediction.

Mobility also introduces distribution shift. A model trained at one location, altitude, band, sensor, or observation period may become inaccurate as the environment changes. Incremental and domain-generalization methods have begun to address spectrum drift [46,47], but monitoring and updating add computation, storage, communication, and energy costs.

Relevant resource constraints include processor capacity, memory, inference latency, update time, communication overhead, sensing energy, battery capacity, payload, and thermal limits. A model with short inference latency may still require expensive adaptation, and a low parameter count does not guarantee low latency on hardware that does not efficiently support the model operations.

Accordingly, this review distinguishes three forms of efficiency:

- Data efficiency: Labeled or target-domain data required for training or adaptation;
- Model efficiency: Parameters, model size, computational complexity, memory, and inference latency;
- System efficiency: Sensing overhead, communication cost, update cost, power consumption, energy use, and mission-level effects.

These dimensions are complementary rather than interchangeable. Reducing the number of sensing operations does not establish that the prediction model is lightweight, and reducing model size does not necessarily reduce the total system energy if frequent updating or communication is required.

### 3.4. Development of UAV-Oriented Spectrum Prediction

Conventional spectrum-prediction studies mainly modeled temporal, frequency, or spatial correlations at fixed sensing locations [1,2,3,4]. UAV-oriented research extended this setting to future locations and mobile paths.

The early UAV-oriented studies considered next-location spectrum-state prediction using HMM-based methods and location-related information [6,7]. The two-dimensional HMM extension introduced time–frequency modeling [8]. Nonlinear homotopy estimation was subsequently used for spectrum-state duration prediction [10], and homotopy-informed recurrent learning extended the approach to LSTM-based prediction [9]. The arbitrary-flight-path method further generalized the spatial transfer procedure to multiple unknown positions within a predefined area [11].

Additional Core studies investigated UAV communication spectrum prediction, three-dimensional spatial prediction, low-altitude intelligent networks, multi-UAV distributed learning, and future hopping-pattern prediction [12,13,14,15,16,17,18]. The UAV may serve as a communication platform in some studies, as the RF source or prediction target in others, and as a sensing or computing node in distributed-learning settings.

A separate research direction used CNN-LSTM, recurrent, or reinforcement-learning models to predict RF sequences and frequency-hopping behavior associated with UAVs [15,16,17,18,19]. These studies are relevant to UAV RF awareness, but they should not be interpreted as evidence that a moving UAV can predict the spectrum available for its own communication.

UAV-adjacent studies investigated three-dimensional sensing, active radio-map estimation, volumetric measurements, compressed tensor completion, ROI-driven deployment, cooperative spectrum sensing, opportunistic access, and altitude-dependent spectrum activity [20,21,22,23,24,25,26,27,28,29,30]. These studies provide evidence concerning data acquisition, spatial sampling, altitude variation, and deployment constraints, but they were not counted as direct future-prediction studies unless future prediction was an explicit task.

Overall, UAV-oriented spectrum prediction developed from next-location state prediction toward duration, arbitrary-path, three-dimensional, low-altitude, sparse-data, distributed, and frequency-hopping prediction. Representative direct UAV and low-altitude evidence is summarized in Table 2. At the same time, label-efficient learning, efficient inference, and drift adaptation were mostly investigated in separate radio-spectrum settings. The principal unresolved issue is that UAV-oriented prediction, target-domain adaptation, efficient inference, and onboard hardware validation have rarely been evaluated jointly.

## 4. Label-Efficient Learning Methods

Few-shot learning and label-efficient learning provide important approaches for alleviating the shortage of labeled samples. The following subsections systematically review four types of methods: data augmentation, transfer learning and domain adaptation, meta-learning, and self-supervised and semi-supervised learning. Their applicable conditions and inherent limitations in UAV spectrum prediction scenarios are also analyzed.

### 4.1. Data Augmentation

Data augmentation increases the effective diversity of training data by transforming existing observations or generating new samples. In spectrum prediction, augmentation may involve segmentation, perturbation, synthetic signal generation, or time–frequency image synthesis.

GAN-based studies combined synthetic-data generation with cross-band spectrum prediction [32,35]. One study used GSM900 uplink data as the source domain and HF measurements in the 20–30 MHz range as the target domain. The target set contained 128 real images, while 4000 generated images were used for pretraining [32]. The results support the use of generated samples for pretraining under limited target-domain data, but the reported limitations included blurred generated images, source–target distribution mismatch, and inadequate representation of anomalous frequency points.

For UAV applications, generative augmentation is potentially useful when real-flight measurements are scarce. Its effectiveness depends on whether the generated data preserve characteristics associated with altitude, position, mobility, interference, antenna configuration, and frequency band. A larger synthetic dataset does not by itself demonstrate improved representation of the target UAV environment.

Generated data should therefore be evaluated using an independent real-data test set. Distribution-level similarity or visual quality is insufficient unless it improves future-prediction performance on real target-domain observations. Current evidence supports generative augmentation as a data-efficiency technique, but direct validation with UAV-acquired target-domain data remains limited.

### 4.2. Transfer Learning and Domain Adaptation

Transfer learning reuses knowledge learned in a source domain for a target domain [31,32,33,34,35]. Domain adaptation is a more specific case in which the method explicitly addresses differences between source and target distributions. In spectrum prediction, domains may differ in frequency band, sensing station, geographic location, service type, sensor, or measurement condition.

Cross-band transfer learning has been investigated using LSTM, temporal–frequency fusion, and other deep architectures [31,34]. The reported studies indicate that transfer can be useful when target-domain data are limited, but that the benefit decreases or disappears as the target dataset becomes sufficiently large. GAN-assisted transfer methods further generated target-like samples before prediction-model training [32,35].

A transfer- and meta-learning framework adapted a ResNet model using 50 target-domain samples [33]. This supports small-sample cross-band adaptation, although the study was not conducted with UAV-acquired target-domain data. The value of the reported 50-sample setting should not be generalized to a standardized UAV few-shot protocol.

Cross-domain knowledge distillation transferred information from a data-rich station to a data-limited station [39]. Its objective was to improve target-domain prediction rather than to reduce the size of the student model. The reported 5-, 20-, and 50-sample or future-prediction settings should therefore not be interpreted automatically as standardized 5-shot, 20-shot, or 50-shot learning.

For UAV spectrum prediction, transfer learning is plausible only when the source and target domains share sufficiently stable temporal, frequency, spatial, or propagation structure. Changes in frequency band, altitude, trajectory, geographic area, antenna configuration, propagation conditions, and interference may cause substantial distribution shift. Source–target similarity and adaptation cost should therefore be reported together with prediction results.

### 4.3. Few-Shot and Meta-Learning

Few-shot learning refers to adaptation using a small labeled support set followed by evaluation on a separate query set. A small dataset or ordinary fine-tuning procedure should not automatically be classified as few-shot learning.

Meta-learning learns an adaptation strategy across multiple tasks. In spectrum prediction, a task may correspond to a frequency band, location, sensing station, altitude range, or radio environment. The cross-band transfer and meta-learning study used 50 target-domain samples and provided evidence for small-sample adaptation [33]. However, it did not use UAV-acquired target-domain data and did not establish a common UAV support–query protocol.

Homotopy-based parameter transfer constructs a model for an unknown or intermediate location using information from known locations [6,7,8,9,10,11]. This reduces location-specific training requirements but is not equivalent to meta-learning unless the method is explicitly optimized across a distribution of adaptation tasks.

The value of meta-learning for UAV prediction depends on whether meta-training tasks represent the variation expected in frequency, location, altitude, mobility, and interference. The support-set acquisition and labeling costs must also be reported. The current evidence supports further investigation but does not establish superiority of meta-learning for real UAV spectrum prediction.

### 4.4. Self-Supervised and Semi-Supervised Learning

Self-supervised learning constructs supervisory signals from unlabeled observations. Potential pretext tasks for spectrum data include masked time–frequency reconstruction, temporal-order prediction, consistency learning, graph-based representation learning, and physical-field reconstruction.

Joint tensor-structural regularization combines spectrum cartography and prediction using self-supervised information [37]. Graph-structure contrastive learning has also been used for spectrum prediction [40]. FieldFormer reconstructs physical fields through tensor attention priors [41], while an untrained deep prior addresses domain-factored spectrum cartography without conventional supervised training [42]. Federated radio-map estimation further explores distributed learning across multiple sensing nodes [43].

These studies indicate that unlabeled or incompletely observed measurements can support representation learning and spatial reconstruction. Radio-map reconstruction and future spectrum prediction should nevertheless remain separate in the synthesis. Evidence from map reconstruction supports spatial representation or observation completion, but it does not directly establish future temporal prediction unless a forecasting task was explicitly evaluated.

The final evidence set also included reinforcement-learning, physics-informed, and diffusion-based prediction methods [45,47,48]. These methods exploit interaction feedback, distributed observations, physical constraints, or probabilistic generation, but they should not automatically be described as label-free. Reinforcement learning depends on reward or interaction feedback, while federated and diffusion-based methods may still require supervised target information.

For UAV systems, self-supervised pretraining is attractive because unlabeled spectrum observations may be easier to acquire than verified future-state labels. Its usefulness should be established through downstream prediction performance under unseen locations, altitudes, trajectories, dates, and interference conditions.

### 4.5. Relevance to UAV Spectrum Prediction

The reviewed label-efficient methods address different components of the UAV data problem. Generative augmentation increases the amount of training material; transfer learning reuses information from related domains; meta-learning seeks rapid adaptation from a small support set; self-supervised methods exploit unlabeled observations; semi-supervised learning combines limited labeled data with larger unlabeled collections; and incremental or drift-aware methods address changes after deployment [31,32,33,34,35,36,37,38,39,40,41,42,43,44,45,46,47,48].

**Figure 4:**
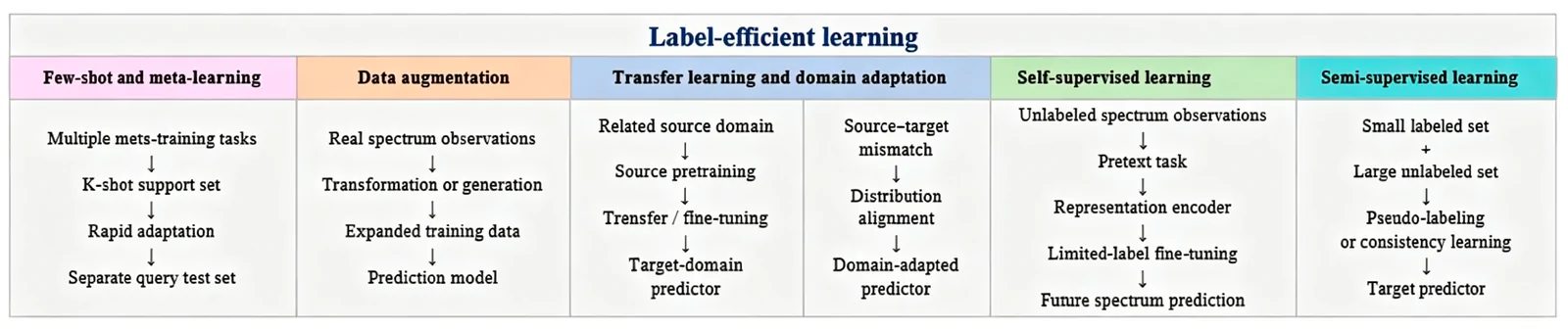
Conceptual organization of label-efficient learning methods and their potential roles in UAV spectrum prediction.

The relationships among these method categories and their potential roles in UAV spectrum prediction are summarized in Figure 4. A focused comparison of representative label-efficient and cross-domain studies is provided in Table 3.

These mechanisms are complementary but not interchangeable. A method developed for cross-band terrestrial data may be useful when the UAV target domain shares stable spectrum structure with the source domain, but it may fail under changes in altitude, trajectory, geographic area, antenna configuration, or interference. Similarly, a method that reduces target-data requirements may remain unsuitable for onboard use if adaptation requires excessive computation, memory, or communication.

The evidence also reveals a distinction between reducing data requirements and reducing deployment cost. Cross-domain distillation and homotopy-based transfer may reduce target-domain training or location-specific retraining, but they do not necessarily produce a smaller or faster inference model. Conversely, a compressed model may reduce inference cost without solving the lack of target-domain data.

The methods shown in Figure 4 should therefore be evaluated along at least two separate axes: the reduction in labeled-data requirements and the cost of adaptation or inference. A method may be data-efficient but computationally expensive, or lightweight during inference but dependent on substantial target-domain labeling.

For UAV applications, the most promising direction is coordinated design of data acquisition, domain adaptation, uncertainty estimation, and deployment. Such a design should be validated using real UAV target-domain data and should report both post-adaptation prediction performance and the cost of obtaining and applying the adaptation.

## 5. Trade-Offs Between Few-Shot Generalization and Lightweight Deployment

The relevance of a method to onboard prediction should not be confused with actual onboard UAV validation. The reviewed evidence is therefore discussed separately in terms of model efficiency, adaptation efficiency, embedded or edge-device implementation, and onboard flight validation.

### 5.1. Lightweight Network Architectures

A lightweight model should be supported by evidence such as reduced parameter count, model size, computational complexity, memory use, inference latency, or energy consumption. Fast training, reduced sensing frequency, and reduced location-specific retraining represent different forms of efficiency and should not be used as direct evidence of model compactness.

The reviewed studies used dictionary learning, recurrent-model complexity analysis, fast LSTM initialization, broad learning, graph learning, online sequential learning, feature selection, and composite two-dimensional LSTM architectures [51,52,53,54,55,56,57,60,61,62,63,64,65,66,67].

Dictionary-learning methods provide low-dimensional representations for spectrum prediction [51]. LSTM-based studies analyzed performance and complexity, while improved initialization reduced training time [52,53]. These results support computational or adaptation efficiency but do not automatically establish low memory or low energy on UAV processors.

The adaptive broad learning network used pseudoinverse-based learning and incremental nodes [56]. It reported CPU training times of 0.7594–1.3594 s and inference times of 0.0029–0.0099 s. Because parameter count, FLOPs, memory, and energy were not reported, this study provides evidence of fast CPU execution rather than a complete quantitative assessment of model lightweightness.

Lightweight continuous-time graph learning combined sparse graph attention and continuous-time recurrent modeling [58]. It reported reductions in CPU processing time and memory usage in the evaluated setting, but a complete parameter and FLOP analysis was not available in the supplied extraction. Its results should therefore be interpreted as platform-specific computational evidence.

Cloud–edge–UAV architectures can move pretraining or model updating away from the UAV [59]. This may reduce onboard computation but introduces communication latency, connectivity dependence, synchronization cost, and additional system-level energy consumption. The benefit therefore depends on the operating environment and cannot be inferred from the division of computation alone.

### 5.2. Pruning and Quantization

Pruning removes redundant parameters or structures from a trained model. Unstructured pruning removes individual weights, whereas structured pruning removes channels, filters, layers, or blocks. Quantization reduces the numerical precision of model weights, activations, or both.

The extracted evidence did not provide sufficient direct results to regard pruning or neural-network quantization as validated solutions for UAV spectrum prediction. These methods are therefore better presented as potential deployment techniques than as established findings of the reviewed UAV prediction literature.

Input-data quantization should also be distinguished from model quantization. Quantizing sensed spectrum values or applying adaptive decision thresholds does not demonstrate low-bit quantization of model parameters or activations.

Direct quantization and pruning evidence for future spectrum prediction remains limited in the reviewed literature. Therefore, lightweight spectrum-prediction studies were mainly assessed through model-level complexity, training and inference time, memory usage, and hardware implementation evidence where available.

Future compression studies should compare original and compressed models in terms of prediction performance, parameter count, model size, FLOPs or MACs, peak memory, inference latency, power, and energy per inference. Robustness after compression should also be assessed under changes in SNR, frequency band, location, trajectory, and prediction horizon.

### 5.3. Knowledge Distillation

Knowledge distillation transfers information from a teacher model to a student model, but its purpose differs across studies.

Compression-oriented distillation aims to reduce the size or computational cost of the student model while retaining teacher performance [55]. This claim requires direct comparisons of teacher and student parameters, model size, FLOPs or MACs, latency, memory, energy, and prediction accuracy.

Cross-domain distillation transfers knowledge from a data-rich domain to a data-limited domain [39]. Its main objective is target-domain adaptation rather than model compression. Because parameter or computational reductions were not established in the supplied evidence, it should not be treated as evidence of lightweight deployment.

Incremental or bidirectional distillation may help retain previously learned information during online updating [46]. In this case, the relevant issues are update time, stored-data requirements, forgetting, and performance under distribution drift.

A large teacher may be trained in the cloud or at an edge server, while a smaller student performs inference on the UAV. Nevertheless, onboard suitability can be claimed only after the student has been evaluated on the intended UAV hardware and under representative flight conditions.

### 5.4. Deployment-Oriented Evaluation

The reviewed studies provide evidence at different deployment levels. A detailed comparison of lightweight and deployment-oriented studies is summarized in Table 4. Algorithm-level studies report computational time or update efficiency without using resource-constrained hardware. Embedded or edge-device studies execute the model on platforms such as the Raspberry Pi. Onboard UAV validation requires execution on UAV-mounted hardware under flight conditions and should not be inferred from either of the former categories.

A prediction-based spectrum-sensing framework was implemented on a Raspberry Pi 5 with two ADALM-PLUTO SDRs [49]. The reported shortest end-to-end system cycle was 28.101 ms, the prediction thread required 27.799 ms, and the average power consumption was approximately 4.8 W. This result demonstrates embedded edge-device implementation. Energy-efficient duty-cycle control has also been studied from a primary-user-awareness perspective, but this line of work supports system-level energy management rather than direct future spectrum prediction [44].

An LSTM-based RF spectral-prediction study reported a real-time FPGA implementation [50]. FPGA execution provides relevant evidence for deterministic and accelerated inference, but it does not by itself establish UAV-mounted or in-flight validation.

The protocol-aware UAV RF study reported 12.7 million parameters and 24.7 ms inference time for a CNN-LSTM on an RTX2080Ti [19]. Because other models in the comparison had fewer parameters or shorter inference times under some conditions, the CNN-LSTM should be described as a model offering a particular performance–complexity balance rather than as the lightest model.

The adaptive broad learning network provides evidence of fast CPU training and inference [56], whereas the continuous-time graph model provides platform-specific timing and memory results [58]. These studies are useful for identifying possible efficiency mechanisms, but their hardware settings and reporting conventions do not support direct cross-study ranking.

A complete deployment assessment should connect model-level measurements with system-level outcomes. In addition to parameters, FLOPs or MACs, memory, latency, and power, future work should examine sensing overhead, communication cost, update frequency, energy per access decision, spectrum-access reliability, and flight-time impact.

## 6. Synthesis, Research Gaps, and Future Directions

### 6.1. Cross-Study Synthesis and Evidence Gaps

The reviewed literature shows that UAV-oriented spectrum prediction has expanded from next-location and temporal prediction to more complex settings involving time–frequency dynamics, spectrum-state duration, arbitrary flight paths, three-dimensional space, low-altitude environments, and distributed learning [6,7,8,9,10,11,12,13,14,15,16,17,18,19]. Some studies have also considered RF activity emitted by or associated with UAVs. However, this task should be distinguished from predicting the spectrum available to a moving UAV, as the two involve different prediction targets and operational purposes.

Research on transfer learning, generative augmentation, meta-learning, self-supervised learning, drift adaptation, model simplification, and embedded inference provides useful methodological support for addressing limited labeled data and constrained computational resources [31,32,33,34,35,36,37,38,39,40,41,42,43,44,45,46,47,48,49,50,51,52,53,54,55,56,57,58,59,60,61,62,63,64,65,66,67,76]. Studies of UAV spectrum measurement, mapping, mobility, and propagation conditions further clarify the characteristics of the aerial operating environment [20,21,22,23,24,25,26,27,28,29,30]. Nevertheless, these research streams have largely developed separately. Evidence obtained from simulations, fixed terrestrial sensors, laboratory RF recordings, or embedded edge devices supports the corresponding experimental settings, but does not by itself establish generalization or onboard feasibility under real flight conditions.

Direct comparison across studies is also limited by differences in prediction tasks, datasets, partition strategies, definitions of limited-data learning, evaluation metrics, and hardware platforms. Consequently, the available evidence does not support a universal ranking of methods. A more defensible conclusion is that UAV-oriented prediction, data-efficient learning, and deployment-oriented efficiency have each received substantial attention, whereas their integration and validation within a unified real-flight system remain insufficient.

### 6.2. Generalization and Deployment Efficiency

Generalization and deployment efficiency should be regarded as coupled design objectives rather than inherently conflicting properties. Models with greater capacity may provide stronger representations but impose higher memory and computational costs. Compact models are more suitable for local inference, yet excessive simplification may reduce prediction accuracy or adaptation capability. Transfer learning, knowledge distillation, model compression, and online adaptation can alter this balance, but their effectiveness depends on the target task and deployment context.

The appropriate trade-off is influenced by source–target similarity, the amount and representativeness of target-domain data, spectrum and trajectory drift, prediction horizon, adaptation frequency, and the capabilities of the target processor. Moreover, efficiency cannot be inferred from a single indicator: fewer parameters do not necessarily imply lower latency or energy consumption on a specific platform, and shorter training time does not demonstrate suitability for onboard inference. The current literature therefore does not identify a universally optimal architecture. Model selection should instead seek a task-specific balance among prediction reliability, adaptation cost, latency, memory use, and energy consumption.

### 6.3. Future Directions

Future research should first strengthen the empirical foundation of UAV spectrum prediction through representative flight data and consistent evaluation protocols. Datasets should reflect variations in mobility, altitude, frequency, location, and propagation conditions, while evaluation should examine generalization across environments rather than performance under random data splits alone. For limited-data settings, comparisons among transfer learning, domain adaptation, meta-learning, and conventional fine-tuning should be conducted under consistent target-domain conditions.

A second direction is to improve adaptation to dynamic aerial environments. UAV motion, propagation characteristics, and spectrum variation should be incorporated into prediction models in a principled manner. In addition, online adaptation should respond to meaningful distribution shifts rather than update continuously, thereby balancing performance recovery against computational cost and the risk of catastrophic forgetting. Existing drift-aware and physics-informed studies provide useful foundations [46,47,48,64], but their effectiveness still requires validation with UAV-acquired data.

Future studies should also treat computational efficiency as a system-level requirement. Model compression and lightweight architectures should be designed for specific onboard hardware, while cloud–edge–UAV collaboration may distribute training, adaptation, and inference across different computing nodes [59]. The suitability of these approaches should be assessed in terms of their overall effects on prediction performance, latency, memory, communication, and energy consumption, rather than through a single efficiency indicator.

Ultimately, progress in this field depends on end-to-end validation under representative flight conditions. Future systems should integrate target-domain adaptation, efficient inference, uncertainty estimation, and spectrum-access decisions on UAV-mounted hardware. Such validation is necessary to determine whether improvements at the algorithmic level can translate into reliable spectrum decisions and acceptable resource consumption during actual UAV operation.

## 7. Conclusions

This review examined the development of UAV spectrum prediction from temporal and next-location prediction toward time–frequency, duration, trajectory-aware, three-dimensional, and UAV-associated RF prediction. The synthesis showed that most existing methods had been evaluated using fixed terrestrial, simulated, or mixed radio-spectrum datasets. Their ability to generalize across UAV trajectories, altitudes, locations, frequency bands, and propagation conditions therefore remained insufficiently established.

The review distinguished strict few-shot learning from the broader category of label-efficient learning. Transfer learning provided the most consistent evidence for adaptation when related source-domain data were available, whereas generative augmentation, self-supervised learning, meta-learning, and cross-domain knowledge distillation offered complementary mechanisms for reducing dependence on labeled target-domain data. However, evidence obtained under limited terrestrial data did not establish few-shot generalization in dynamic UAV environments.

The review also differentiated data efficiency, model efficiency, adaptation efficiency, and system efficiency. Existing studies had investigated lightweight architectures, online updating, model compression, FPGA acceleration, and edge-assisted processing. These studies demonstrated different forms of efficiency, but faster training, lower adaptation cost, reduced model size, and shorter inference latency were not equivalent. Embedded and FPGA implementations indicated practical potential, although they did not constitute end-to-end onboard validation under representative flight conditions.

Overall, the evidence supported interpreting label-efficient generalization and lightweight deployment as a conditional design trade-off rather than an inherent conflict. The principal gap identified by this review was the limited integration of UAV-acquired data, target-domain adaptation, efficient inference, uncertainty-aware decision making, and onboard hardware validation within a single operational framework. Representative flight datasets, standardized limited-data protocols, drift-aware adaptation, hardware-aware optimization, and end-to-end evaluation should therefore receive greater attention in future research.

## Figures and Tables

**Table 1:** Final evidence classification.

Evidence Category	Final Records	Final Citation Numbers	Main Use
Core/E1	14	[6,7,8,9,10,11,12,13,14,15,16,17,18,19]	Direct UAV or low-altitude prediction evidence
Adjacent-A/E2	38	[31,32,33,34,35,36,37,38,39,40,41,42,43,44,45,46,47,48,49,50,51,52,53,54,55,56,57,58,59,60,61,62,63,64,65,66,67,76]	Transferable radio-spectrum prediction and deployment evidence
Adjacent-B/E3	11	[20,21,22,23,24,25,26,27,28,29,30]	UAV sensing, mapping, sampling, and scenario constraints
Background/E4	13	[1,2,3,4,5,68,69,70,71,72,73,74,75]	Terminology, reviews, and methodological context
Total retained	76	[1,2,3,4,5,6,7,8,9,10,11,12,13,14,15,16,17,18,19,20,21,22,23,24,25,26,27,28,29,30,31,32,33,34,35,36,37,38,39,40,41,42,43,44,45,46,47,48,49,50,51,52,53,54,55,56,57,58,59,60,61,62,63,64,65,66,67,68,69,70,71,72,73,74,75,76]	Narrative synthesis
Superseded versions	4	Not retained	Version control
Duplicate aliases	3	Not separately counted	Deduplication
Task-mismatched exclusions	4	Not retained	UAV detection, classification, tracking, or localization

**Table 2:** Direct UAV and low-altitude spectrum-prediction evidence.

Reference	Prediction Target	Data and Setting	Main Method	Reported Result	Evidence Boundary
Zhao et al. [6]	Next-location spectrum state	Simulation and ground RTL-SDR measurements; approximately 12.28 MHz; seven positions; 300-sample training sequence	SNS-HMM	Simulation prediction probability up to 72.12% and 66.54%; measured results 84.67–99.5%	No real UAV-flight acquisition; hardware NR
Luo et al. [7]	Next-time and next-location spectrum state	Ground measurements approximating approximately 100 m flight altitude; 12.2792 MHz; two known and five unknown positions	HT-HMM	Prediction probability 85–95%; average running time 0.0155 s	Ground-based measurement and CPU execution; no onboard validation
Zhang et al. [8]	Time–frequency spectrum state	MATLAB Poisson simulation; 5×100 input matrix; 18 km path	HT-2DHMM	Approximately 2–3 percentage-point improvement over 1D-HMM	No real spectrum measurement; training/test split NR
Chen et al. [9]	Next-location spectrum state	Ground RTL-SDR measurements near Chengdu airport; direct-path setting	HT-LSTM	HT-LSTM 72.00–82.83%; direct LSTM 80.03–88.38%	Limited to the interpolation setting; hardware metrics NR
Luo et al. [10]	Spectrum-state duration	Ground RTL-SDR measurements; 122.792 MHz and other frequency points; 1200 samples per position	NLH-HMM	Prediction Feasibility Ratio 54–70%	Feasibility ratio is not classification accuracy; no onboard validation
Dai and Huang [12]	UAV communication spectrum state	UAV communication scenario; detailed quantitative fields NR	RF-spectrum prediction method	NR in supplied extraction	Direct UAV relevance, but quantitative evidence incomplete
Cheng et al. [13]	Three-dimensional spatial spectrum	ElectroSense measurements; 600–700 MHz; four ground sensors and 100 frequency points	3DS-FF and MS-TE	MAPE 1.04%; MAE 0.0754; RMSE 0.0904	Fixed ground sensors; no airborne acquisition or onboard hardware
Luo et al. [11]	Arbitrary-path spectrum state	Ground RTL-SDR measurements; 124.8411 MHz; three known and 14 unknown positions	MOHT-HMM	82.49–93.90%; HMM 84.05–94.60%	Arbitrary path remained simplified; no in-flight validation
Zhang et al. [14]	Multi-UAV frequency-hopping prediction	Distributed UAV learning scenario; detailed quantitative fields NR	Incremental learning	NR in supplied extraction	UAV role includes sensing and computation; onboard evidence NR
Deng et al. [15]	Nonlinear hopping-frequency prediction	Navigation-confrontation scenario	HT-HMM	NR in supplied extraction	RF or navigation scenario; deployment evidence NR
Zhao et al. [16]	Low-altitude spectrum prediction	Low-altitude intelligent-network scenario	Meta-BAGRU	NR in supplied extraction	UAV-related application, but target-domain and hardware details NR
Li et al. [17]	FHSS short-term prediction	FHSS signal scenario	Temporal-attention CNN	NR in supplied extraction	UAV/RF-source relevance; onboard execution NR
Tuong et al. [18]	Future hopping points	RF frequency-hopping scenario	Deep reinforcement learning	NR in supplied extraction	UAV-associated RF prediction; onboard execution NR
Basak et al. [19]	Future time–frequency sequence of UAV RF signals	MATLAB synthetic data and four commercial UAV RF recordings; 2.4 GHz; X310 SDR	CNN-LSTM and YOLO-assisted models	93.2% at 1 MHz and 99.5% at 5 MHz; CNN-LSTM inference 24.7 ms	UAV is the RF target/source, not the prediction platform; no onboard validation

**Table 3:** Focused comparison of label-efficient and cross-domain methods.

Reference	Mechanism	Target-Domain Setting	Quantitative Information	Evidence Boundary
Lin et al. [31]	Cross-band deep transfer learning	GSM1800 to GSM900 or TV-band data; target data reported by days	One-day target setting favored transfer; exact label count NR	Non-UAV terrestrial evidence
Lin et al. [32]	GAN augmentation and transfer	GSM900 uplink to HF 20–30 MHz; 128 real target images and 4000 generated images	Approximately 0.1 s/epoch; RMSE and FID used	Generated-data mismatch and anomalous-frequency limitations
Peng et al. [33]	Transfer learning and meta-learning	Cross-band target adaptation using 50 target samples	Small-sample adaptation reported	Not a standardized UAV few-shot protocol
Li et al. [34]	Temporal–frequency fusion with transfer learning	Related radio-spectrum domains	Target-domain performance improvement reported	No UAV-acquired target-domain data
Peng et al. [35]	GAN-based data conversion	Cross-band spectrum prediction	Generated data used for target-domain augmentation	Transferability to low-altitude flight conditions NR
Pan et al. [36]	Deep stacked autoencoder and long-term prediction	Real-world spectrum data	Long-term prediction evaluated; detailed target-data protocol NR	Unsupervised representation evidence, not UAV onboard validation
Chen et al. [37]	Tensor-structural self-supervised regularization	Joint spectrum cartography and prediction	Quantitative fields NR in supplied extraction	Spatial reconstruction and prediction must remain separated
Pan et al. [38]	Deep 3D pyramid vision transformer	Multidimensional spectrum prediction	Quantitative fields NR in supplied extraction	Transferable 3D evidence; UAV hardware NR
Shang et al. [39]	Cross-domain knowledge distillation	Data-rich Station A to data-limited Station B	MSE improvement of 45.9% over CESP in one 50-sample setting; 39.4% over DSIL in another setting	Not compression-oriented distillation
Li et al. [40]	Graph contrastive learning and multiscale fusion	Multichannel spectrum prediction	Quantitative fields NR	Non-UAV graph-learning evidence
Chen et al. [41]	Self-supervised physical-field reconstruction	Physical-field completion	Quantitative fields NR	Reconstruction evidence, not necessarily future prediction
Timilsina et al. [42]	Untrained deep prior	Domain-factored spectrum cartography	Quantitative fields NR	No UAV onboard validation
Zhang et al. [43]	Federated radio-map estimation	Distributed sensing nodes	Quantitative fields NR	Supports distributed spatial estimation rather than direct future prediction

**Table 4:** Focused comparison of lightweight and deployment-oriented studies.

Reference	Efficiency Mechanism	Parameters	Latency or Timing	Memory and Power	Hardware	Deployment Level
Kim and Giannakis [51]	Dictionary learning	NR	NR	NR	NR	Algorithm-level
Radhakrishnan et al. [52]	LSTM performance–complexity analysis	NR	NR	NR	NR	Algorithm-level
Radhakrishnan and Kandeepan [53]	Fast LSTM initialization	NR	Training efficiency reported	NR	NR	Algorithm-level
Mosavat-Jahromi et al. [54]	Statistical and neural prediction modeling	NR	NR	NR	NR	Algorithm-level
Cheng et al. [55]	Compression-oriented knowledge distillation	Teacher–student comparison reported; exact parameters NR in supplied extraction	Hardware latency NR	Memory and energy NR	General computing platform	Model-compression evidence
Ji et al. [56]	Adaptive broad learning and incremental nodes	NR	Training 0.7594–1.3594 s; inference 0.0029–0.0099 s	NR	Intel Xeon Silver 4210R CPU	CPU efficiency
Ji et al. [57]	Generative augmented cascade broad learning	NR	NR	NR	NR	Algorithm-level
Li et al. [58]	Sparse continuous-time graph learning	NR	GPU and CPU timing reported	Memory reported as 1.42 GB; energy NR	RTX2080Ti and CPU	Platform-specific computational evidence
Cheng et al. [59]	Cloud–edge two-timescale learning	NR	NR	Communication and energy NR	Cloud/edge setting	System architecture evidence
Mahboob et al. [60]	Graph-based recurrent prediction	NR	Real-time objective; exact value NR	NR	NR	Algorithm-level
Zou and Wang [61]	High-resolution spectrum mobility modeling	NR	NR	NR	NR	Algorithm-level
Zou and Wang [62]	Efficient high-resolution spectrum prediction	NR	NR	NR	NR	Algorithm-level
Wang et al. [63]	Online sequential extreme learning	NR	Online update setting	NR	NR	Online algorithm evidence
Li et al. [64]	Robust online spectrum-map prediction	NR	Online prediction setting	NR	NR	Online algorithm evidence
Al-Tahmeesschi et al. [65]	Feature-based DNN simplification	NR	NR	NR	NR	Algorithm-level
Ozyegen et al. [66]	Memory-impact analysis	NR	NR	Memory considered; exact values NR	Land-mobile-radio setting	Complexity evidence
Aygül et al. [67]	Composite 2D-LSTM	NR	NR	NR	General computing platform	Multidimensional prediction evidence
Rojas et al. [49]	Prediction-assisted sensing	Partial; YOLOv8n 3 M parameters	System cycle 28.101 ms; prediction thread 27.799 ms; YOLO 0.208 ms	Approximately 4.8 W estimated; peak memory NR	Raspberry Pi 5 and two ADALM-PLUTO SDRs	Embedded edge validation
Siddhartha et al. [50]	FPGA LSTM implementation	NR	Real-time implementation reported	NR	FPGA	Hardware acceleration
Basak et al. [19]	CNN-LSTM RF prediction	12.7 M parameters	24.7 ms on RTX2080Ti	Memory and energy NR	RTX2080Ti and X310 SDR	Workstation and laboratory RF evidence

## Data Availability

The original contributions presented in this study are included in the article. Further inquiries can be directed to the corresponding author.

## Abbreviations

The following abbreviations are used in this manuscript:

UAV	Unmanned aerial vehicle
RF	Radio frequency
PSD	Power spectral density
RSS	Received signal strength
HMM	Hidden Markov model
CHMM	Continuous hidden Markov model
2DHMM	Two-dimensional hidden Markov model
HT-HMM	Homotopy estimation based hidden Markov model
MOHT-HMM	Multi-order homotopy estimation based hidden Markov model
NLH-HMM	Non-linear homotopy estimation based hidden Markov model
LSTM	Long short-term memory
CNN	Convolutional neural network
CNN-LSTM	Convolutional neural network-long short-term memory
GRU	Gated recurrent unit
TCN	Temporal convolutional network
DNN	Deep neural network
GNN	Graph neural network
MAML	Model-agnostic meta-learning
NAS	Neural architecture search
SDR	Software-defined radio
RTL-SDR	RTL software-defined radio
FPGA	Field-programmable gate array
CPU	Central processing unit
GPU	Graphics processing unit
MACs	Multiply-accumulate operations
FLOPs	Floating-point operations
MSE	Mean squared error
MAE	Mean absolute error
RMSE	Root mean squared error
MAPE	Mean absolute percentage error
FID	Frechet inception distance
SNR	Signal-to-noise ratio
FHSS	Frequency-hopping spread spectrum
YOLO	You only look once
PRISMA	Preferred Reporting Items for Systematic Reviews and Meta-Analyses
PRISMA-ScR	Preferred Reporting Items for Systematic Reviews and Meta-Analyses extension for Scoping Reviews
PRISMA-S	Preferred Reporting Items for Systematic Reviews and Meta-Analyses literature search extension
INT8	8-bit integer
NR	Not reported
